# To jump or not to jump - The Bereitschaftspotential required to jump into 192-meter abyss

**DOI:** 10.1038/s41598-018-38447-w

**Published:** 2019-02-19

**Authors:** M. Nann, L. G. Cohen, L. Deecke, S. R. Soekadar

**Affiliations:** 1 Clinical Neurotechnology Laboratory, Department of Psychiatry and Psychotherapy, Neuroscience Research Center (NWFZ), Charité – University Medicine Berlin, Berlin, Germany; 2 Applied Neurotechnology Laboratory, Department of Psychiatry and Psychotherapy, University Hospital of Tübingen, Tübingen, Germany; 3 National Institute of Neurological Disorders and Stroke (NINDS), Human Cortical Physiology and Stroke Neurorehabilitation Section, Bethesda, Maryland USA; 4 Department of Clinical Neurology, Medical University Vienna, Wien, Austria

## Abstract

Self-initiated voluntary acts, such as pressing a button, are preceded by a surface-negative electrical brain potential, the Bereitschaftspotential (BP), that can be recorded over the human scalp using electroencephalography (EEG). While the BP’s early component (BP1, generated in the supplementary and cingulate motor area) was linked to motivational, intentional and timing properties, the BP’s late component (BP2, generated in the primary motor cortex) was found to be linked to motor execution and performance. Up to now, the BP required to initiate voluntary acts has only been recorded under well-controlled laboratory conditions, and it was unknown whether possible life-threatening decision making, e.g. required to jump into a 192-meter abyss, would impact this form of brain activity. Here we document for the first time pre-movement brain activity preceding 192-meter bungee jumping. We found that the BP’s spatiotemporal dynamics reflected by BP1 and BP2 are comparable before 192-meter bungee jumping and jumping from 1-meter. These results, possible through recent advancements in wireless and portable EEG technology, suggest that possible life-threatening decision-making has no impact on the BP’s spatiotemporal dynamics.

## Introduction

The decision to perform a self-initiated act, such as releasing an arrow during Kyūdō (Japanese art of archery), pushing off for the first glide in alpine (downhill) skiing or jumping from a rock in cliff diving, requires integration and synchronization of internal and external sensory information^1^, as well as a sense for the opportune moment for action, a moment termed καιρός in Ancient Greek (e.g. Ilias IV, 185)^2^. In Greek mythology, καιρός, the youngest son of Zeus, was worshiped as a deity depicted as a young man with winged feet holding a Libra in one hand to weigh the right moment in time and a knife in the other hand to cut off ties to the past. Having only some tuft of hair on the front head while being bald on his occiput and constantly whirling around, one had to seize the right moment to grasp him by his tuft, or the right moment would be missed, possibly for ever.

The notion that success of a purposeful, self-initiated complex action depends on the right timing has its analogy in findings showing that perceptual sensitivity in the visual and auditory domain depends on brain oscillatory phase angles at which stimuli are either optimally processed or hampered. While the neural substrates of exteroceptive and interoceptive processes, such as visual and auditory perception or common sensation (“Gemeingefühl”)^3^, were extensively studied^4^, the neural substrate underlying the sense for the opportune moment for action involving integration of extero- and interoceptive processes are still widely unknown. Developing a sense for the opportune moment for a self-initiated act in a particular context (e.g., in platform or cliff diving competitions) often requires many years of training, particularly when motor initiation involves possible life-threatening decision making.

For a long time, the neural origins of self-initiated acts remained an enigma. While neurophysiological experimentations up to the 1960ies were mainly influenced by behaviourism focusing on stimulus-response paradigms, the discovery of the Bereitschaftspotential (BP) in 1964 (engl. readiness potential)^5,6^ signified an entirely new direction in neurophysiological research investigating the neural substrate of *self-initiated* voluntary acts, i.e. acts that are not triggered by external stimuli.

**Figure 1:**
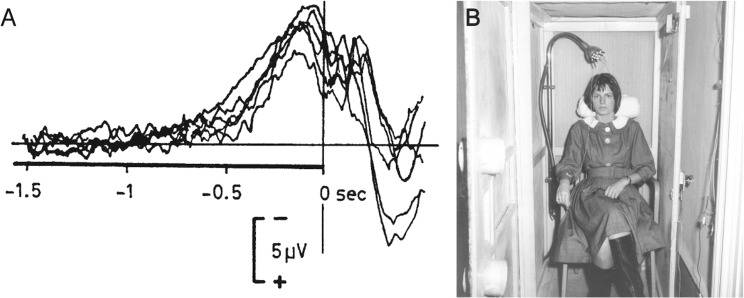
(**A**) Reverse-computation („Rückwärtsanalyse“) of electroencephalographic (EEG) signals before self-initiated voluntary finger movements (right index finger flexion) evidenced a surface-negative potential shift beginning approximately 1.5 s before finger movement-related electromyographic (EMG) activity could be detected. This characteristic EEG signal was termed „Bereitschaftspotential“ (BP) (in engl. also readiness potential) by Kornhuber and Deecke^5^ and provided first important insights to the neural origins of self-initiated acts^5,6^ (superposition of 6 experiments of the same participant at 6 different days with 250 trials each, i.e. 1500 self-initiated finger movements (right index finger flexion) of the same participant B.L., adapted from Deecke, *et al*.^39^). (**B**) Participants had to sit still in a Faraday cage with the head reclined into a headrest to avoid EEG artefact (photograph from the original experimental setup used in Kornhuber and Deecke^5^, provided by Lüder Deecke, Austria).

Due to the non-stationary and dynamic nature of brain activity, stimulus-response paradigms usually require averaging of brain activity over multiple trials. This can be easily achieved by using the stimulus as the common starting point (trigger) for averaging. Due to lack of a known starting point, however, such approach cannot be applied to unpredictable self-initiated movements. Building on early EEG systems that used ferromagnetic tapes to store the recorded signals, an important technological innovation and prerequisite for the discovery of the BP was to reverse such tapes and use the onset of electromyographic (EMG) activity related to a volitional act, e.g. moving a finger, as marker for reverse computation (“Rückwärtsanalyse”) of the EEG signal^7^. In the original experiment, EEG signals of approximately 250 trials were averaged and evidenced a surface-negative electric potential building up approximately 1.5 s before the onset of EMG activity (Fig. 1A). Study participants had to sit still in a Faraday cage with their head reclined into a headrest to avoid EEG artefacts (Fig. 1B). The recorded brain potential was most evident at the vertex electrode (i.e. at electrode position Cz according to the international 10/20 convention) and reached amplitudes of up to 10–15 μV^5,6^. Based on the waveform and involved generators, two phases or components of the BP can be distinguished: the “early BP” (BP1) generated by the pre-supplementary motor area (pre-SMA), SMA and lateral premotor cortex bilaterally that begins about 1.5 s before movement onset, and the “late BP” (BP2) with a steep surface-negative slope generated by the primary motor cortex (M1) and lateral premotor cortex with precise somatotopy that begins approximately 400 ms before movement onset^8,9^. Due to the somatotopic organization of the cortex, the BP of bilateral feet movements, e.g. during jumping, is usually not lateralized showing its maximum amplitude at Cz^10^.

Using a multimodal neuroimaging approach based on functional magnet resonance imaging (fMRI) and electroencephalography (EEG), Cunnington *et al*.^11^ showed a specific correlation between global electrical field power of pre-movement neural activity recorded at Cz and the metabolic (i.e. blood-oxygen-level dependent, BOLD) activity of the anterior mid-cingulate cortex (aMCC). By applying dynamic causal modelling, they found strong reciprocal interactions between the aMCC and the supplementary motor areas (SMA) that they identified to be important for the sustained activity during the early BP (BP1). Moreover, besides involvement of the cortico-basal ganglia-thalamo-cortical loop (motor loop), a number of studies indicate that the prefrontal cortex (PFC), a region that was found to be tightly linked to the concept of willpower^12^ (i.e. the capacity to control one’s own thoughts and actions, as well as the ability to override an unwanted thought, feeling or impulse), decision making and executive control^13^, plays a critical role for initiation of voluntary movements^14^. Particularly the medial part of the PFC (mPFC) was shown to be important for regulating neuronal circuits linked to fear and anxiety^15^.

While it was shown that the magnitude and waveform of the BP depend on various factors, such as force exerted, speed and precision of movement as well as pace of movement repetitions or complexity of movement^16^, it was unknown whether the BP also underlies movement initiation in possible life-threatening decision making, such as self-initiating a jump into a 192-meter abyss. The main reason for this gap in knowledge is that, despite numerous replications and several thousand publications since its discovery, the BP has never been recorded in a real-life situation outside the laboratory where motor initiation in possible life-threatening decision making can be investigated.

Moreover, it was unknown whether the affective anticipation associated with the decision to self-initiate a jump into a 192-meter abyss impacts the BP’s spatiotemporal dynamics. A number of studies showed that affective anticipation is signified by increased fronto-central surface-negativity^17,18^ found to be generated in the insular cortex^19^. This fear-induced surface-negativity, however, involved anticipation of an external stimulus, for instance anticipation of a mild electric shock^17^. Brain electric activity preceding *self-initiated* movements involving fearful and potentially life-threatening decision making was not studied yet.

Today, more than 50 years after the discovery of the BP, EEG technology has improved across multiple domains, particularly in terms of miniaturization, digitization, signal quality and wireless signal transmission. These advancements fostered the development of innovative neurotechnologies, such as brain-machine interfaces (BMIs) that translate brain activity into control commands of external machines, robots or computers. Recently, it was shown, for example, that severely paralyzed quadriplegics can operate an EEG-controlled brain/neural hand-exoskeleton (B/NHE) to perform activities of daily living, such as eating and drinking in an outside restaurant^20^. We thus reasoned that, under optimal conditions, recording of the BP outside the laboratory might be feasible, even in extreme real-life scenarios such as 192-meter bungee jumping.

Based on the model that the BP results from an unequal ratio of surface-positive and surface-negative potential shifts accumulating towards a decision threshold^21,22^, a threshold at which initiation of muscle contractions is inevitable (the “point-of-no-return” found to be reached approximately 200 ms before detection of electromyographic activity)^23,24^, we expected that affective anticipation related to possible life-threatening decision making should not impact the BP’s spatiotemporal dynamics reflected by BP1 and BP2. We thus hypothesized that the onset of BP1, the maximal amplitude of BP2 as well as waveforms of BP1 and BP2 at Cz reflecting the BP’s spatiotemporal dynamics are comparable before 192-meter bungee jumping and jumping from 1-meter.

Deriving from an ancient ritual on Vanuatu, an island in the South Pacific Ocean, bungee jumping, i.e. jumping from a tall structure while fixed to an elastic cord, has become a popular and commercialized activity with millions of jumps since the 1980ies. Besides representing a test of courage (bungee jumping was seen as an expression of boldness in ancient Vanuatu), it was shown that bungee jumping can result in a marked increase of euphoria ratings and concentration of beta-endorphin (by more than 200%) measured immediately after a bungee jump^25^. The risk associated with bungee jumping, which was estimated to be lower than the risk involved in riding a bicycle or dancing^26^, stands in stark contrast to the bungee jumper’s fear and subjective impression that jumping from such height is potentially life threatening.

Here, we report successful recording of the BP across two semi-professional cliff divers who performed several bungee jumps from a 192-meter bungee platform (the second highest bungee platform in Europe). To evaluate the impact of possible life-threatening decision making on the BP’s spatiotemporal dynamics, such as onset, maximal amplitude and waveform of components, BPs recorded before bungee jumping were compared to BPs recorded before the same participants jumped from 1-meter.

## Results

### Surface-negative EEG deflection before self-initiated 192-meter bungee jumping

**Figure 2:**
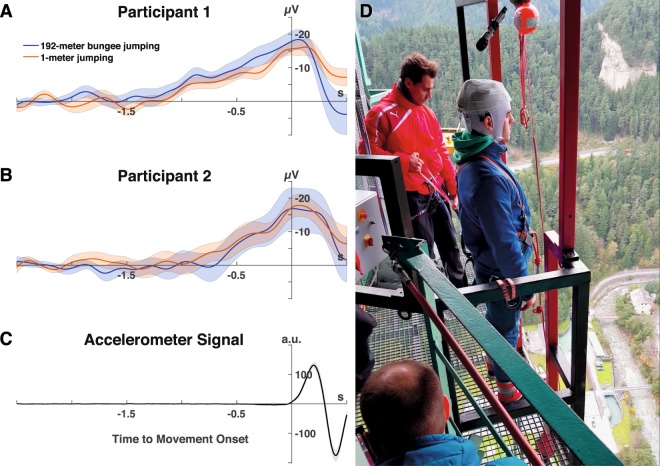
Electroencephalographic (EEG) recordings before 192-meter bungee jumping (blue) and before jumping from 1-meter (orange) evidenced a surface-negative potential shift over the vertex electrode (Cz) with the characteristic features of the Bereitschaftspotential (BP) (**A**) participant 1, (**B**) participant 2; average over all trials). The standard error is illustrated as orange and blue shaded areas, respectively. Please note that the BP has been plotted with the y-axis inverted for easier comparison with the BP illustrated by Deecke, *et al*.^39^ (Fig. 1A). (**C**) Movement onset before bungee jumping was detected by an accelerometer integrated into the EEG system. The solid line shows the averaged accelerometer signal across all trials of both jumpers. The standard error is indicted by the shaded area. Reverse-computation of the pre-bungee jumping BPs was time-locked to the detected movement onset. (**D**) One of the semi-professional cliff divers in pre-bungee jumping posture. 192-meter bungee jumps were initiated by coming up on the toes and bending forward. The EEG system was attached to the jumper’s occiput using a customized electrode cap (EasyCap^®^, Herrsching, Germany), adhesive tape and an elastic net dressing.

Analysis of EEG recordings at Cz showed a surface-negative electrical trend beginning approximately 1.5 s and a clear surface-negative deflection 400 ms before movement onset of self-initiated 192-meter bungee jumping (Fig. 2A,B) evidencing successful detection of BP (Table 1) (participant 1: mean EEG deflection (±standard error): −11.63 ± 2.71 µV, *t*(14) = −4.29, *p* < 0.001, *d* = 1.11; participant 2: −8.37 ± 3.86 µV, *t*(11) = −2.17, *p* = 0.027, *d* = 0.63).

**Table 1:** Surface-negative electroencephalography (EEG) deflection 400 ms before movement onset. P1 = participant 1, P2 = participant 2, # jumps = number of jumps, *M* = mean, *SE* = standard error, *t* = t-value, *df* = degrees of freedom, significance levels: *p* < 0.05*, *p* < 0.01**, *p* < 0.001***, *d* = Cohen’s *d*.

	jump height (m)	# jumps (included)	M (µV)	SE (µV)	One-tailed paired-sample t-test		
					*t (df)*	*p*	*d*
P 1	192	16 (15)	−11.63	2.71	−4.29 (14)	<0.001***	1.11
	1	16 (16)	−8.53	1.68	−5.08 (15)	<0.001***	1.27
P 2	192	14 (12)	−8.37	3.86	−2.17 (11)	0.027*	0.63
	1	16 (14)	−9.08	3.70	−2.45 (13)	0.015*	0.65

### Spatiotemporal dynamics recorded before 192-meter bungee jumping vs. 1-meter jumping

**Figure 3:**
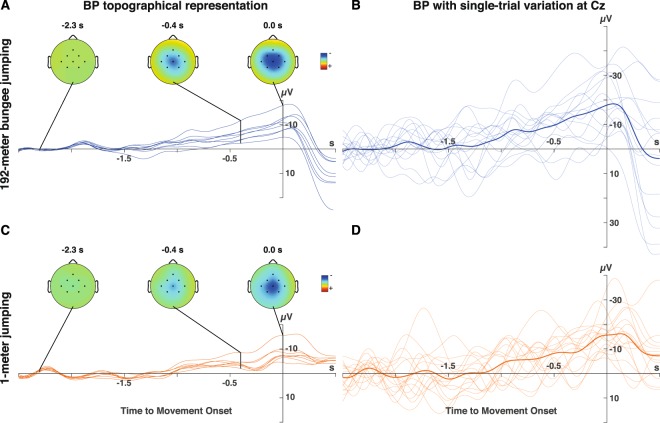
Left panels: Averaged EEG signals over all trials of all 8 recorded electrodes during 192-meter bungee jumping (**A**) and during 1-meter jumping (**C**). BP’s spatiotemporal dynamics of participant 1 are visualized with topographical plots at selected latencies: at −2.3 s representing the reference range, at −0.4 s characterising the transition point between ‘early’ (BP1) and ‘late BP’ (BP2), and at movement onset (0.0 s). Right panels: BP with single-trial EEG variation of participant 1 at Cz during 192-meter bungee jumping (**B**) and during 1-meter jumping (**D**). The averaged EEG signals (resulting BPs) are indicated by solid lines whereas the variation of single-trial EEGs is visualised by transparent lines.

Analysis of EEG recordings at Cz 400 ms before movement onset of self-initiated 1-meter jumps showed a clear surface-negative deflection (Table 1) (participant 1: mean EEG deflection (± standard error): −8.53 ± 1.68 µV, *t*(15) = −5.08, *p* < 0.001, *d* = 1.27; participant 2: −9.08 ± 3.70 µV, *t*(13) = −2.45, *p* = 0.015, *d* = 0.65). Visualization of the recorded BP’s spatiotemporal dynamics with topographical representations at selected latencies (average over all trials) showed a characteristic surface-negative deflection without any lateralization due to the bilaterality of the jumping movement (Fig. 3A,C) (Movie 1).

**Figure 4:**
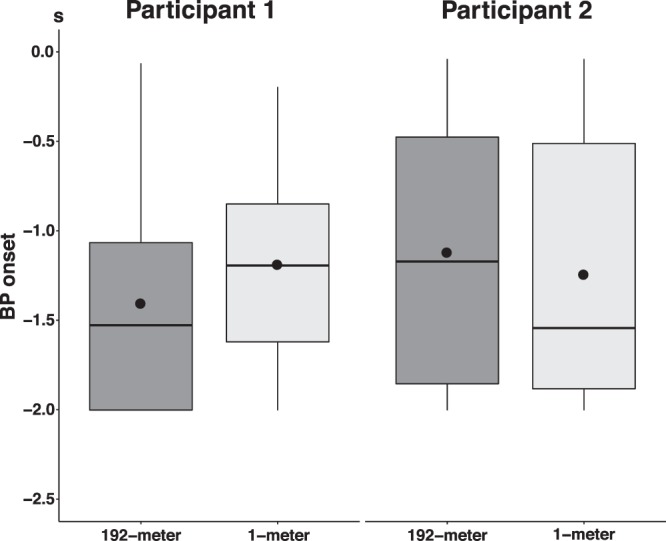
Differences between BP onset recorded at Cz before 192-meter bungee jumping and jumping from 1-meter for both participants. No differences were found between jumping heights. Centerlines of boxplots show the median, while dots show the mean. Box limits indicate the 25^th^ and 75^th^ percentiles.

Comparison of BP onset of BPs recorded before 192-meter bungee jumping (participant 1: median (*Mdn*) = −1.53 s, interquartile range (*IQR*) = 0.94 s; participant 2: *Mdn* = −1.17 s, *IQR* = 1.38 s) and BPs recorded before 1-meter jumping (participant 1: *Mdn* = −1.19 s, *IQR* = 0.77 s; participant 2: *Mdn* = −1.54 s, *IQR* = 1.37 s) showed no difference (participant 1: *U* = 93, *p* = 0.29, *r* = 0.19; participant 2: *U* = 94, *p* = 0.62, *r* = 0.10) (Table 2, Fig. 4).

**Table 2:** BP onset. P1 = participant 1, P2 = participant 2, *Mdn* = median, *IQR* = inter-quartile range, *U* = U-value, significance levels: *p* < 0.05*, *p* < 0.01**, *p* < 0.001***, *r* = Wilcoxon’s *r*.

	192-meter bungee		1-meter		Mann-Whitney-U test		
	Mdn (s)	IQR (s)	Mdn (s)	IQR (s)	*U*	*p*	*r*
P 1	−1.53	0.94	−1.19	0.77	93	0.29	0.19
P 2	−1.17	1.38	−1.54	1.37	94	0.62	0.10

**Figure 5:**
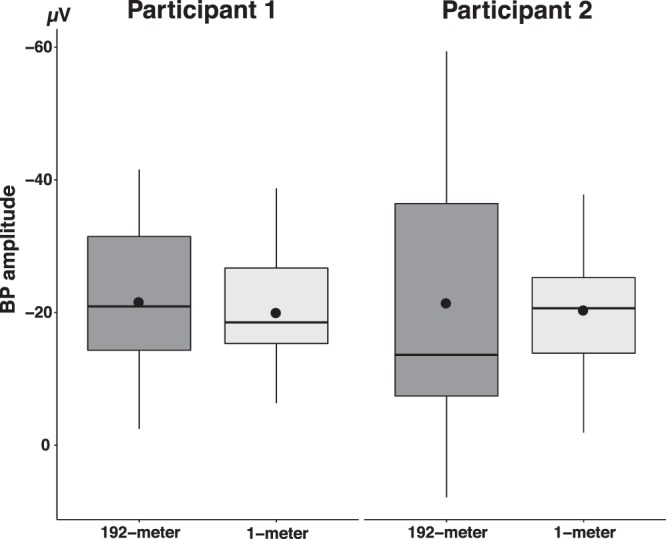
Differences between maximal BP amplitude recorded at Cz before 192-meter bungee jumping and jumping from 1-meter for both participants. No differences were found between jumping heights. Centerlines of boxplots show the median, while dots show the mean. Box limits indicate the 25^th^ and 75^th^ percentiles.

Similarly, maximal amplitudes of BPs recorded before 192-meter bungee jumping (participant 1: mean amplitude (± standard error): −21.61 ± 2.89 µV; participant 2: −21.44 ± 6.17 µV) and before 1-meter jumping (participant 1: −19.99 ± 2.13 µV; participant 2: −20.37 ± 2.93 µV) were comparable (participant 1: *t*(29) = −0.46, *p* = 0.65, *d* = 0.16; participant 2: *t*(16) = −0.16, *p* = 0.88, *d* = 0.07) (Table 3, Fig. 5).

**Table 3:** Maximal BP amplitude. P1 = participant 1, P2 = participant 2, *M* = mean, *SE* = standard error, *t* = t-value, *df* = degrees of freedom. In case of unequal sample size, a two-sample t-test was used. Significance levels: *p* < 0.05*, *p* < 0.01**, *p* < 0.001***, *d* = Cohen’s *d*.

	192-meter bungee		1-meter		Two-sample t-test		
	M (µV)	SE (µV)	M (µV)	SE (µV)	*t (df)*	*p*	*d*
P 1	−21.61	2.89	−19.99	2.13	−0.46 (29)	0.65	0.16
P 2	−21.44	6.17	−20.37	2.93	−0.16 (16)	0.88	0.07

Calculating Pearson’s correlation coefficient between BP waveforms preceding 192-meter bungee jumping and 1-meter jumping evidenced highly correlated BP components for each participant (participant 1: BP1: *r* = 0.98, *p* < 0.001, BP2: *r* = 0.97, *p* < 0.001; participant 2: BP1: *r* = 0.88, *p* < 0.001, BP2: *r* = 0.97, *p* < 0.001) (Table 4) indicating that possible life-threatening decision-making does not impact BP’s spatiotemporal dynamics recorded at Cz.

**Table 4:** BP waveform correlation for ‘early’ (BP1) and ‘late BP’ (BP2) components. *r* = Pearson’s r correlation coefficient, significance levels: *p* < 0.05*, *p* < 0.01**, *p* < 0.001***.

		Pearson’s r	
		*r*	*p*
P 1	BP1	0.98	<0.001***
	BP2	0.97	<0.001***
P 2	BP1	0.88	<0.001***
	BP2	0.97	<0.001***

## Discussion

To our knowledge, this is the first report on successful recordings of the BP outside the laboratory and in the context of self-initiating a voluntary act in possible life-threatening decision making. We found that the BP can be successfully detected by averaging EEG data from less than 16 trials documenting feasibility of such recordings outside the laboratory. We found that BP onsets and maximal amplitudes recorded before self-initiated 192-meter bungee jumps and before 1-meter jumps were comparable. High correlation between BP waveforms recorded before 192-meter bungee jumping and 1-meter jumping suggests that possible life-threatening decision making has no impact on the BP’s spatiotemporal dynamics.

Catalysed by Benjamin Libet’s experiments in the 1980ies^27^ indicating that the conscious decision for a self-initiated act occurs not earlier than 200–250 ms before the onset of EMG activity, the discovery of the BP led to a vivid and controversial discussion about free will^28^ (it should be noted that neither Kornhuber and Deecke^29^ nor Libet ever called human freedom or free will into question^30^. While Kornhuber and Deecke admit that “absolute freedom from nature is an impossibility”^29^ leaving humans relative freedom, or freedom in degrees, Libet argued that, even if a volitional process is initiated unconsciously, the conscious function can still control the outcome by exerting a *veto*). While it is unlikely that neurophysiological experiments can decisively contribute to solving this controversy, feasibility of BP recordings in real-life scenarios allow now for investigating the neural mechanisms underlying self-initiated voluntary acts under realistic conditions. In this context, EEG recordings using a larger number of electrodes (≥32) and application of advanced signal processing techniques, such as connectivity^14^ or entropy measures^31,32^, may provide important insights regarding the involved brain networks and their interactions. Moreover, implementation of real-time signal processing within the framework of brain-computer interfaces (BCIs) allows answering some profound research questions, for example determining the point in time at which people are still able to cancel (“veto”) a movement after the elicitation of a BP^23,24^. Moreover, establishing BCIs based on motor-related cortical potentials (MRCP)^33^ that include the BP to detect movement intentions in everyday life environments may enhance existing BCI systems, e.g. to restore activities of daily living to quadriplegics^20^ or in the context of neurorehabilitation^34^.

The fact that emotional anticipation or fear did not impact the BP’s onset and amplitude suggests that BP might be particularly suitable to detect movement preparation even in emotionally engaging situations. Besides further substantiating that neurotechnologies, i.e. technological tools to interact with the brain, are now about to enter everyday life environments^35^, our report shows that brain physiological assessments previously regarded unfeasible, e.g. assessment of the BP outside the laboratory, are viable and can extend the possibility to test scientific hypotheses that were previously un-testable.

It could be argued that, in most lab-based investigations, decision-making, i.e. the decision to obey the instruction to self-initiate a particular movement, e.g. to press a button, was already concluded before the beginning of the actual experiment. If so, participants have only to decide *when* and *how* to press and not *whether* to press. In bungee jumping or other extreme activities that involve possibly life-threatening decision making, this decision-making process cannot be assumed to be concluded at any point in time before the actual jump. Many bungee jumping novices interrupt their first attempt and need an external trigger to overrule their instinct not to jump. Even bungee jumpers with year-long experience and dozens of jumps report that each jump requires them to overcome this instinct suggesting a decisive role of the PFC coordinating neuronal circuits related to willpower, executive functions and fear responses. While our data indicate that this coordination process does not influence the onset or waveform of the pre-bungee jumping BP, the limited number of electrodes and trials did not allow for analyses of fronto-parietal cortico-cortical interactions. Further studies are needed to elucidate these possible network interactions and their relationship to the generation of the BP. Moreover, it would be interesting to also evaluate post-movement brain potentials and their components^9^, an endeavour particularly challenging due to movement-related EEG artefacts (e.g. related to eye movements or muscle activity^5^).

The neurophysiological data reported here were recorded from semi-professional cliff divers who have never engaged in bungee jumping before. Both participants reported that they had a very strong inner resistance to jump before each trial suggesting that no relevant desensitization due to over-familiarity occurred. Due to the necessary averaging of EEG signals, however, a possible desensitization or habituation effect cannot be fully ruled out, and it is conceivable that neurophysiological data recorded from individuals who are not accustomed to high altitudes or individuals with acrophobia show different results. While the necessarily low number of trials demonstrates that the pre-bungee jumping BP is very well expressed, the small number of participants and trials constrains the possibility to exclude habituation effect and to generalize these findings towards other real-life scenarios. In this context, use of virtual reality (VR) technology may be particularly helpful as a complementary approach, not only to increase the number of trials and validate generalizability of the reported results, e.g. towards other real-life scenarios, but also to further investigate the neural origins of self-initiated acts in a controlled environment.

## Methods

### Experimental setup

Two semi-professional cliff divers (both males, 19 years each) who have never engaged in bungee jumping performed self-initiated voluntary jumps from two different heights: first outside the laboratory from a 192-meter bungee jumping platform (Fig. 2D) (operator: Rupert Hirner Bungee Jumping GmbH, Europa Bridge in Innsbruck, Austria; http://www.europabruecke.at/) and second under well-controlled laboratory conditions from 1-meter. Before 192-meter bungee jumping, both participants were informed by the platform operator that they were free to jump as often as they liked, and that all jumps were entirely voluntary and could be aborted at any time. Each participant performed up to 16 jumps from both heights (Table 1).

Before the jumps, specific instructions on how to perform the jumps without generating excessive muscle artefacts while generating a clear trigger signal for reverse computation were provided (e.g. keeping head motions and blinking to a minimum, relaxing the arms and trunk, initiating the jump by coming up on the toes and bending forward). Besides allowing for precise detection of movement onset due to an initial upward movement, muscle artefacts were kept at a minimum by initiating the jump with the toe extensors (the most distant muscles from the EEG recording sites).

### Portable electroencephalography (EEG) with built-in accelerometer

For EEG recordings, a wireless and portable 8-channel EEG system (LiveAmp^®^, Brain Products GmbH, Gilching, Germany) in combination with an active electrode system (actiCAP^®^, Brain Products GmbH) was used. Use of active electrodes, i.e. electrodes with build-in readout circuitry that locally amplify and buffer EEG signals before transmitting the signals through cablings, ensured that the signal in the cables was insensitive to interference, e.g. due to motion^36^. EEG was recorded from eight conventional recording sites (Fz, FC1, FC2, C3, Cz, C4, CP1, CP2 according to international 10/10 system). Ground and reference electrodes were placed at Fpz and the mastoid, respectively. Impedance of all electrodes was kept below 5 kΩ to ensure high EEG signal quality. Since the onset of self-initiated movements is unpredictable, a built-in three-axis acceleration sensor (±2 g) provided a clear trigger signal for reverse computation. Unlike other possible movement detection methods, e.g. measuring electromyography (EMG) from leg muscles, the applied method reduced the necessary equipment and cabling to a minimum. The EEG amplifier and acceleration sensor [dimensions (w × d × h): 83 × 51 × 14 mm, overall weight: approx. 60 g (incl. built-in battery)] were fixed to the jumper’s occiput using customized electrode caps (EasyCap^®^, Herrsching, Germany), adhesive tape and an elastic net dressing. Besides allowing for EEG recordings for up to 3 hours, the system transmitted the recorded signals wirelessly via Bluetooth to a signal processing unit (Sony Vaio Duo 13^®^ equipped with an Intel Core i7^®^ processor).

### Signal processing

EEG and accelerometer data were sampled at 250 Hz and band-pass-filtered at 0.1 to 3 and 5 Hz, respectively. Based on studies indicating that a band-pass filter with a lower edge of 0.1 Hz (corresponds to a time constant of 1.6 s) yields the best detection accuracy for movement related cortical potentials (MRCPs, a group of brain potentials that includes BP)^37^, the EEG band pass-filter was set to 0.1–3 Hz. EEG signals recorded from Cz before each jump (defined as trial) were time locked to the onset of an accelerometer signal exceeding two standard deviations recorded during movement preparation at rest (defined as detected movement onset) (Fig. 2C) and then epoched into 3-second windows (for illustration of single-trial EEG signals at Cz recorded before 192-meter bungee jumping and before 1-meter jumping see Fig. 3B,D). Epochs ranged from −2.5 s to +0.5 s with a baseline correction relative to the first 0.5 s (reference window: −2.5 s to −2.0 s). Epochs with non-physiological signal amplitudes exceeding ±100 µV or large drifts before detected movement onset were excluded from further analysis. Topographical plots of the averaged EEG signals were visualized at selected latencies (−2.3 s, −0.4 s and 0.0 s).

### Outcome measures and statistics

As suggested by Shibasaki and Hallett^9^, successful detection of a BP was defined as surface-negative deflection of the EEG signal 400 ms before movement onset relative to a mean reference value recorded at −2.5 s to −2.0 s across all trials. To test whether there was a difference in the BP’s spatiotemporal dynamics between 192-meter bungee jumping and 1-meter jumping, averaged BP onsets, maximal BP amplitudes and BP waveforms were compared. BP onset was defined as the time point at which the EEG signal showed a continuous surface-negative deflection without further zero-crossing for more than 500 ms occurring after the reference window (−2.0 s) and movement onset (0.0 s). Maximal BP amplitude was defined as the maximal surface-negative deflection at movement onset ±100 ms. While the BP’s early component (BP1) was defined as surface-negative EEG deflection from −1.5 s to −0.4 s, the BP’s late component (BP2) was defined as surface-negative EEG deflection from −0.4 s to 0.0 s.

High comparability of averaged BP waveforms was defined as a Pearson’s r correlation coefficient higher than 0.6 (i.e. strong correlation). Normality of data was tested using a Shapiro-Wilk test. Provided normality of data, parametric Student’s t-tests were performed (one-tailed paired-sample t-tests as well as two-sample t-tests). In case normality of data could not be assumed, non-parametric Mann-Whitney-U tests were performed. Effect sizes as measured by Cohen’s *d* for parametric tests and Wilcoxon’s *r* for non-parametric tests were calculated^38^. For all tests, significance level was set to *p* ≤ 0.05.

## Supplementary information

BP’s spatiotemporal dynamics

## Data Availability

All datasets are available from the corresponding author on reasonable request.
